# A cryptic promoter in potato virus X vector interrupted plasmid construction

**DOI:** 10.1186/1471-2199-8-17

**Published:** 2007-03-05

**Authors:** Yuyuan Guo, Thomas L German, Ronald D Schultz

**Affiliations:** 1 Department of Pathological Sciences, School of Veterinary Medicine, 2015 Linden Dr. Madison, WI53706, USA; 2 Department of Entomology, College of Agricultural and Life Sciences, University of Wisconsin-Madison, 1630 Linden Dr. Madison, WI53706, USA

## Abstract

**Background:**

Potato virus X has been developed into an expression vector for plants. It is widely used to express foreign genes. In molecular manipulation, the foreign genes need to be sub-cloned into the vector. The constructed plasmid needs to be amplified. Usually, during amplification stage, the foreign genes are not expressed. However, if the foreign gene is expressed, the construction work could be interrupted. Two different viral genes were sub-cloned into the vector, but only one foreign gene was successfully sub-cloned. The other foreign gene, canine parvovirus type 2 (CPV-2) VP1 could not be sub-cloned into the vector and amplified without mutation (frame shift mutation).

**Results:**

A cryptic promoter in the PVX vector was discovered with RT-PCR. The promoter activity was studied with Northern blots and Real-time RT-PCR.

**Conclusion:**

It is important to recognize the homologous promoter sequences in the vector when a virus is developed as an expression vector. During the plasmid amplification stage, an unexpected expression of the CPV-2 VP1 gene (not in the target plants, but in *E. coli*) can interrupt the downstream work.

## Background

Potato virus X (PVX), a potexvirus, is a filamentous rod-shaped virus which contains a single plus-sense RNA molecule. The RNA is capped, polyadenylated, and encodes five open reading frames (ORF)[1]. PVX was designed to express foreign genes[2]. Its coat promoter was duplicated in the vector to drive the foreign ORF transcription. The PVX vector has been used to express foreign genes in many host plants[3], in translation studies[4], and for investigating gene silencing[5]. In the current study, the PVX vector was used to express the gp53 gene of BVDV Singer strain[6] and VP1 of CPV-2[7] for vaccine development in a plant vector. The construction of the PVX vector required the new plasmid to be amplified in *E. coli *before inoculating plants, which would be necessary to construct a plant virus vectored viral genes for a vaccine for these two viruses.

In molecular cloning, plasmids need to be amplified in *E. coli *to produce enough plasmid DNA for molecular manipulation. The foreign gene in the expression vectors is not expressed until it is in a eukaryotic host cell; thus, it was surprising to find, when the plasmid was amplified in *E. coli*, the foreign gene was expressed and it killed the bacteria. Cryptic promoter is the promoter homologous sequence that distribute randomly. It was previously reported that there was a cryptic promoter associated with a toxic gene in potato virus Y (PVY) that interrupted cloning work in *E. coli*[8]. The cryptic promoter in PVX has not been reported before. When the eukaryotic expression vectors are derived from viruses, certain viral sequences might be recognized as promoter elements. We tried to sub-clone two animal viral genes into the PVX vector; however, only the gp53 ORF from BVDV was successfully sub-cloned. The other gene, the CPV-2 VP1, could not be sub-cloned into the PVX vector, probably because it was toxic to *E. coli *when expressed. If VP1 had not been expressed in *E. coli*, the cloning would have been possible and the cryptic promoter in the PVX vector would not have been found, as occurred when the gp53 ORF was sub-cloned in the PVX vector and transcribed. The transcription initiation site was determined with RT-PCR. The cryptic promoter activity from PVX was compared with that of the T7 promoter with Real-time RT-PCR.

## Results and discussion

### Comparison of the PVX cryptic promoter with the conserved prokaryotic promoter

**Figure 1:**
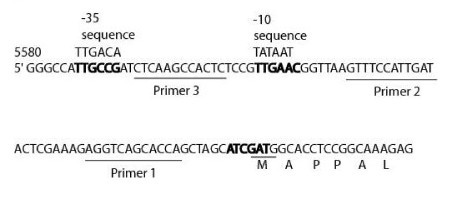
Comparison of the PVX cryptic promoter with the conserved prokaryotic promoter, The bold letters indicate the conserved prokaryotic promoter sequence, -10 and -35 sequences. The cryptic promoter matches 4 of 6 bp in -10 element, and 3 of 6 bp in -36 element. The cryptic promoter sequences are underlined. The bold letters near the start codon (ATG) are the Cla I cloning site. The partial transgene VP1 amino acid sequence MAPPAL is indicated.

The similarity of the end element of the triple block sequences in PVX and the prokaryotic conserved sequence of -10 and -35 were compared (Figure 1). The conserved promoter sequences of prokaryotic organisms are -10 TATAAT and -35 TTGACA, separated by 16–18 base pairs. The prokaryotic homologous element in the PVX vector matched over 50% of the prokaryotic conserved promoter sequence. The homologous element was considered as the cryptic promoter sequence and it was hypothesized to drive the transcription of foreign genes.

### Transcription of foreign genes driven by the cryptic promoter on the PVX vector

Both the BVDV gp53 and the CVP-2 VP1 foreign genes were sub-cloned into the PVX vector. However, only the gp53 ORF in the plasmid could be expressed. In the PVX vector, the cloned VP1 fragment always had a mutation near the start codon (data not shown). We hypothesized that there was a cryptic promoter driving the foreign gene to be transcribed, which lead to expression of a toxic VP1 protein that killed the *E. coli *during plasmid amplification.

**Figure 2:**
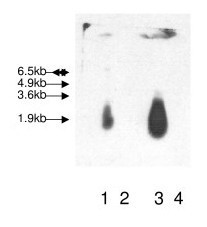
The cryptic promoter in the PVX vector. The transcription of the foreign gene gp53 was detected with Northern blots. Lane 1: 100 ng total RNA from the pgR106gp53 transformed E. coli. Lane 2: 100 ng total RNA of the pgR106 transformed *E. coli *(without foreign gene). The specific gp53 band was detected. Lane 3: 300 ng RNA from the pgR106gp53 transformed *E. coli*. Lane 4: 300 ng total RNA from the pgR106 transformed *E. coli*.

In order to demonstrate the existence of cryptic promoter, *E. coli *was transformed with the PVX vector, pgR106gp53 plasmid. After a four hour culture, the total RNA was isolated. Northern blots showed the foreign gene gp53 was transcribed (Figure 2). With the gp53 specific probe in Northern blots, the pgR106gp53 transformed *E. coli *showing a band of about 1 kb; whereas, the plasmid pgR106-transformed *E. coli *(without the foreign gene) did not. This suggests that cryptic promoter activity existed.

### Determination of the site of transcription initiation

**Figure 3:**
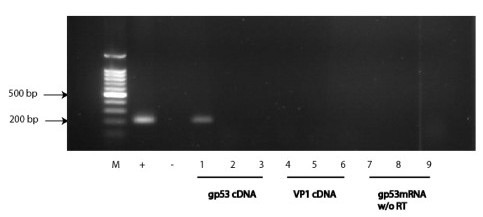
Determination of the initiation of transcription site. Three primers behind the cryptic promoter were designed to run the RT-PCR. Primer 1 was used in Lanes 1, 4, and 7; Primer 2 in Lanes 2, 5, and 8; Primer 3 in Lanes 3, 6, and 9. M stands for the molecular marker. The "+" stands for the positive PCR control. The "-" marker stands for the negative PCR control. Lane 1, Lane 2, and Lane 3 contain the RT-PCR product from pgR106gp53 transformed *E. coli*. Lane 4, Lane 5, and Lane 6 for the RT-PCR product with template RNA from the gpR106VP1 transformed *E. coli*. Lane 7, Lane 8, and Lane 9 for the PCR product with total RNA from the pgR106gp53 transformed E. coli. No PCR product was amplified, showing the RNA did not contain plasmid DNA.

If the hypothesis regarding the cryptic promoter position was correct (Figure 1), the gp53 RNA in *E. coli *should have a 5' untranslated region (5'UTR) from the PVX vector. Based on the sequence behind the cryptic promoter, three different primers in front of the gp53 ORF (Figure 1) and an antisense primer from gp53 sequence (200 bp downstream) were designed to run RT-PCR. Only Primer 1 (nearest to start codon) could amplify the cDNA of gp53. The 5'UTR is about 10 base pairs long (Figure 3). A negative control plasmid (only the vector, without gp53 insertion) transformed *E. coli*, did not have any PCR product, using gp53 specific primer. The mRNA control (isolated RNA without adding reverse transcriptase) showed that the RNA was not contaminated with plasmid DNA because no DNA fragment was amplified. This demonstrated that transcription was initiated at approximately 30 base pairs downstream of the cryptic promoter.

### Comparison of the cryptic promoter with the T7 promoter using Real-time RT-PCR

**Figure 4:**
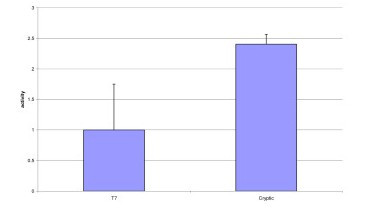
Comparison of the activity of the cryptic promoter and the T7 promoter using Real-time RT-PCR. The average activity of the cryptic promoter (value: 2.4) is higher than that of the T7 promoter (value: 1). The 16S rRNA was the housekeeping gene in this study.

The activity of both the cryptic promoter and T7 promoter (sequence 5' TAATACGACTCACTATAGGGAG) were compared using Real-time RT-PCR. This method uses mathematical formulas to calculate relative expression levels compared to a calibrator. The amount of target gene expression is also normalized to an endogenous housekeeping gene, in this case, 16S rRNA. The cryptic promoter activity was significantly higher than that of the T7 promoter (p < 0.01) (Figure 4).

## Conclusion

Even though the cryptic promoter sequence did not perfectly match the conserved prokaryotic -35 and -10 elements, it matched 50% and 66%, respectively (Figure 1). It has been reported that certain foreign elements can be recognized as a promoter by *E. coli*[9]. Promoter homologous sequences are widely distributed in the genomic sequence of *E. coli*[10]. Cryptic promoters are those sequences that are promoter homologous sequences driving transcription. The cryptic sequences might also interrupt expressions of foreign genes in plants. An example is that the homologous intron sequence within a gene can also interrupt expression of foreign gene[11]. In the current study, a prokaryotic cryptic promoter drove transcription during plasmid amplification. The sequence of the cryptic promoter in PVX is different from that in PVY[8]. The interruption was different because the toxic gene is not from the virus itself as in PVY but from the foreign gene. Cryptic promoters in eukaryotic cells can have different functions, such as increased enzyme expression; tissue-specific or developmental stage-specific gene expression [12-14].

A reporter gene, such as chloramphenicol acetyltransferase (CAT) when sub-cloned behind different promoters could be used to compare the promoter activity[15]. The enzyme expression driven by promoters can reflect the promoters' activities. However, with Real-time RT-PCR, the transcribed RNA could be used as an indicator to show the promoter activity[16]. With real time RT-PCR, the cryptic promoter activity was compared with that of T7 promoter, an inducible promoter.

Although the PVX vector is an expression vector in plants, it needs to be amplified in *E. coli *for molecular manipulation. During the DNA amplifying stage, the foreign genes should not be expressed; however, the foreign genes in the PVX vector were transcribed. If VP1 were not toxic to bacteria its transcription would not influence the cloning work. The failure to sub-clone the VP1 cistron, suggested that VP1 was toxic to bacteria (data not shown). The toxic pressure prevented the non-mutated VP1 from being sub-cloned into the PVX vector.

A number of plant viruses, such as tobacco mosaic virus (TMV) and PVX, have been reported to contain mRNA regions that possess a high degree of homology to both chloroplast rRNA and prokaryotic 16S RNA, which are closely related[17]. It has been demonstrated that a triple block sequence located upstream from the coat protein gene of PVX facilitated the translation of the pokeweed antiviral protein gene in *E. coli *[17]. Even though unexpected expression of a foreign gene can interrupt downstream cloning work, the cryptic promoter makes the PVX vector an expression vector in *E. coli*, able to express foreign genes in both *E. coli *and plants. This ability to express foreign genes either in a prokaryotic organism (*E. coli*) or a eukaryotic organism (plants) makes the PVX vector a potential useful tool to study the differences in transcription and translation between prokaryotic and eukaryotic organisms without changing the vector.

## Methods

### Sub-cloning the foreign genes within the PVX vector

The PVX vector was developed into a useful plant expression vector[2]. PVX vector (pgR106) was used to express the foreign genes. CPV-2 VP1 sub-cloning failed because of a constant frameshift mutation near the start codon (data not shown). The construction of the gp53 ORF within the PVX vector was done to show the cryptic promoter activity. The BVDV (Singer strain) gp53 was previously sequenced[6]. The BVDV Singer-gp53-pGEM plasmid was used as a template for PCR to amplify the gp53 ORF with specific primers (IDT, Coralville, Iowa). The PVX vector contains a ClaI cloning site behind the coat promoter and a Sal I cloning site at the 3'end. The sense primer was 5'-CC**A TCG ****AT**G GAC TTG CAT TGC AAA CCT G-3' with a ClaI restriction enzyme site (bold); the antisense primer was 5'-**GTC GAC **TCA CCC TGA GGC CTT CTG TTC-3' with a Sal I restricted enzyme site (bold). The start codon is underlined. The ClaI sequence was incorporated in front of the start codon.

The PCR reaction conditions were as follows: 4 minutes pre-heating at 95°C, a denaturation step at 95°C for 30 seconds, an annealing step at 55°C for 30 seconds, and a synthesis step at 75°C for 45 seconds. Twenty five cycles of PCR reactions were followed by a seven minute extension reaction at 72°C. The PCR product was then cleaved by ClaI and SalI (Promega, Madison, Wisconsin) in Buffer D. After cleavage, the ORF was ligated with the pgR106 vector.

### Northern blots

A probe for gp53 was made with BioNick DNA labeling system[18]. A biotin label kit (Invitrogen, Carlsbad, California) was used to label the probe for Northern blots detected with chemiluminescent hybridization[19]. The North2South Chemiluminescent hybridization and Detection Kit (Pierce, Rockford, Illinois) was used. The total RNA was isolated with the improved RNA isolation method[20] using RNeasy Plant Mini kit (Qiagen, Valencia, CA) from *E. coli*. The *E. coli *strain JM109 (Promega), was used for transformation with the pgR106gp53 plasmid. The total RNA was isolated from the transformed JM109 and non-insertion plasmid pgR106 plasmid transformed JM109 after culture for 4 hours in Luria-Bertani (LB) medium at 37°C. An amount of 100 ng and 300 ng total RNA from *E. coli *and RNA markers (Promega) were run in a 1% agarose gel in MOPS running buffer at 45 mV for 50 minutes. The gel was then stained with an RNA staining buffer for 15 minutes. The gel was transferred overnight with 20× SSC buffer. The RNA marker was labeled on the nylon membrane after transferring. The membrane was hybridized with a biotin-labeled probe for gp53, following the kit protocol (Pierce).

### RT-PCR

Total cellular RNA was extracted from pgR106gp53 transformed *E. coli *with RNease Mini Kit (Qiagen) and followed the protocol. The gp53 cDNA necessary for the PCR assay was obtained using a cDNA CYCLE KIT (Invitrogen) which is based on a simple and efficient synthesis method[21]. First strand cDNA was synthesized according to the manufacture's instructions by using the antisense primer GCAAGATACCTG from gp53 sequence. Three forward primers were designed from the triple block sequence[22] to locate the transcription of the initiation site, Primer 1 AGGTCAGCACCA, Primer 2 GTTTCCATTGAT, Primer 3 CTCAAGCCACTC (Figure 1) were designed to determine the initiation of the transcription site of the transgene. Primer 1 is the nearest to the transgene followed by Primer 2, then by Primer 3. The primers were synthesized at the UW-Biotechnology Center (Madison, WI).

### Real-time RT-PCR

As CPV-2 VP1 was toxic (data not shown) the cloning work was interrupted by the toxicity and the cryptic promoter. We chose a control expression method employing T7 promoter[23]. By comparing the expression of gp53 under two promoters, the cryptic promoter's activity was compared with T7 promoter activity. A relative quantity of Real-time RT-PCR results were calculated using the comparative threshold cycle (*C*_*T*_) method[24]. The inducer for the T7 promoter was isopropyl β-D-thiogalactoside (IPTG)(GibcoBRL, Gaithersburg, MD). A volume of 1 ml LB medium containing 0.5 μg/ml kanamycin and 1 mM IPTG, was used to culture *E. coli *transformed with both plasmids. The total RNA was isolated with the RNeasy Plant Mini kit (Qiagen, Valencia, CA) for cDNA synthesis. The forward primer GGAAGGATTACTCGCCTGAA from the gp53 ORF was designed to amplify a 200 bp fragment using Primer Express 3.0 software (Applied Biosystems, Foster City, CA). The antisense primer CTCTCGTGCACCTTGGGAGG, a 200 bp sequence downstream of the forward primer, was designed to make cDNA[21] with cDNA CYCLE KIT (Invitrogen). The housekeeping gene was 16S rRNA, as described by Spano[25], using CATGCCGCGTGTATGAAGAA as a forward primer and TCACATCCGACTTGACAGAC as an antisense primer to produce a 200 bp fragment. Briefly, 0.3 μg total RNA recovered from the samples was added to a mixture consisting of magnesium chloride, RNase inhibitor, 10× buffer, oligo-primers, and reverse transcriptase following the kit protocol of the cDNA CYCLE KIT (Invitrogen). The solution was heated at 70°C for 10 minutes, incubated at 45°C for 50 minutes, and finally heated at 95°C for 5 minutes. The cDNA samples were then stored at -20°C for further use. For Real-time RT-PCR experiments, SYBR Green DNA polymerase master mix and 7300 Real-time PCR System (Applied Biosystems) were employed. Briefly 5 μl cDNA was added to a 25 μl Real-time PCR mixture containing 12.5 μl of master mix and 4 μM of each primer. The reaction mix was cycled through the following temperature profile: incubation at 50°C for 2 min and 95°C for 10 min, 40 cycles at 95°C for 20 seconds, one cycle at 60°C for 30 seconds and one cycle at 60°C for 40 seconds. To semi-quantify the promoter activities, an arbitrary threshold was set at cycles 6–15. The ratios of the *Ct*-values of the cryptic promoter and the T7 promoter to the housekeeping gene were determined. Three replications of each promoter activity were done. T-test was employed to determine whether the two promoters' activities were significant different.
